# Harvesting electrical energy from torsional thermal actuation driven by natural convection

**DOI:** 10.1038/s41598-018-26983-4

**Published:** 2018-06-07

**Authors:** Shi Hyeong Kim, Hyeon Jun Sim, Jae Sang Hyeon, Dongseok Suh, Geoffrey M. Spinks, Ray H. Baughman, Seon Jeong Kim

**Affiliations:** 1 Center for Self-powered actuator and Department of Biomedical Engineering, Hanyang University, Seoul, 133-791 South Korea; 2 Department of Energy Science, Sungkyunkwan University, Suwon, Gyeonggido 16419 South Korea; 3 Intelligent Polymer Research Institute, ARC Centre of Excellence for Electromaterials Science, University of Wollongong, Wollongong, New South Wales 2522 Australia; 4 The Alan G. MacDiarmid NanoTech Institute, University of Texas at Dallas, Richardson, TX 75083 USA

## Abstract

The development of practical, cost-effective systems for the conversion of low-grade waste heat to electrical energy is an important area of renewable energy research. We here demonstrate a thermal energy harvester that is driven by the small temperature fluctuations provided by natural convection. This harvester uses coiled yarn artificial muscles, comprising well-aligned shape memory polyurethane (SMPU) microfibers, to convert thermal energy to torsional mechanical energy, which is then electromagnetically converted to electrical energy. Temperature fluctuations in a yarn muscle, having a maximum hot-to-cold temperature difference of about 13 °C, were used to spin a magnetic rotor to a peak torsional rotation speed of 3,000 rpm. The electromagnetic energy generator converted the torsional energy to electrical energy, thereby producing an oscillating output voltage of up to 0.81 V and peak power of 4 W/kg, based on SMPU mass.

## Introduction

The development of practical, cost-effective systems for the conversion of waste heat to electrical energy is an important area of renewable energy research^1–3^. Harvesting energy from low grade waste heat is particularly challenging, since available energy conversion systems, such as thermoelectrics^4,5^ and thermocells^6^, operate most efficiently when exposed to large temperature differences. Other systems that generate electrical energy from time-dependent temperature fluctuations, by using such materials as pyroelectrics^1,7,8^, ferroelectrics^9^, ferromagnets^10–12^, and shape memory metals^13,14^, require large, fast temperature excursions to generate high output power. We here describe novel electromechanical generators driven by thermally powered artificial muscles. The system is shown able to produce useable electrical power from fluctuations in natural convection that occurs in everyday ambient environments.

Our new energy harvesters exploit recently discovered large-stroke tensile and torsional actuators made from twisted and coiled yarns or fibers^15–20^. These artificial muscles produce impressive performance, as demonstrated by coiled nylon fishing line that can generate a peak mechanical power output of up to 50 kW/kg when heated, which is 84 times higher than that of skeletal muscle. Twisted and coiled yarns and fibers exploit twist changes that are driven by yarn or fiber volume changes, which can be induced thermally, photonically, chemically or electrochemically.

We want to harvest useful electrical from very small ambient temperature fluctuations. To do this using the twist-based mechanism of twisted or coiled polymer fibers or yarns requires fibers or yarns having high volumetric thermal expansion coefficients near room temperature. To achieve this goal, we used a commercially available shape memory polyurethane (SMPU) to fabricate torsional artificial muscles that provide large, fast, reversible torsional strokes. We then harvested thermally generated torsional energy of a magnetic rotor driven by a SMPU yarn using an electromagnetic converter of torsional kinetic energy to electrical energy. Using temperature fluctuations of about 4 °C, and a temperature difference between hottest and coldest muscle segments of just 13 °C, the generator produced a peak open-circuit voltage of 0.81 V and a peak electrical power density of 4.0 W/kg (when normalized to muscle weight).

## Results

### Thermal conversation powered torsional SMPU muscle

The SMPU was specifically chosen in order to obtain a torsional muscle that could effectively harvest small temperature fluctuations above ambient temperature as mechanical energy. In particular, the SMPU has a low glass transition temperature (T_g_) of 25 °C and a volumetric thermal expansion coefficient (40 × 10^−5^ m/m·K)^21^ in the target temperature range of 30 °C to 60 °C, which is considerably higher than for other artificial muscle polymers, such as nylon 6,6 (8 × 10^−5^ m/m·K) and polyethylene (11 × 10^−5^ m/m·K)^22^. In addition, the SMPU shows a high degree of strain recovery during a shape memory heat/deform/cool and re-heat cycle^23^. These properties suggest that the SMPU is suitable for reversible torsional muscles driven by small thermal fluctuations.

**Figure 1:**
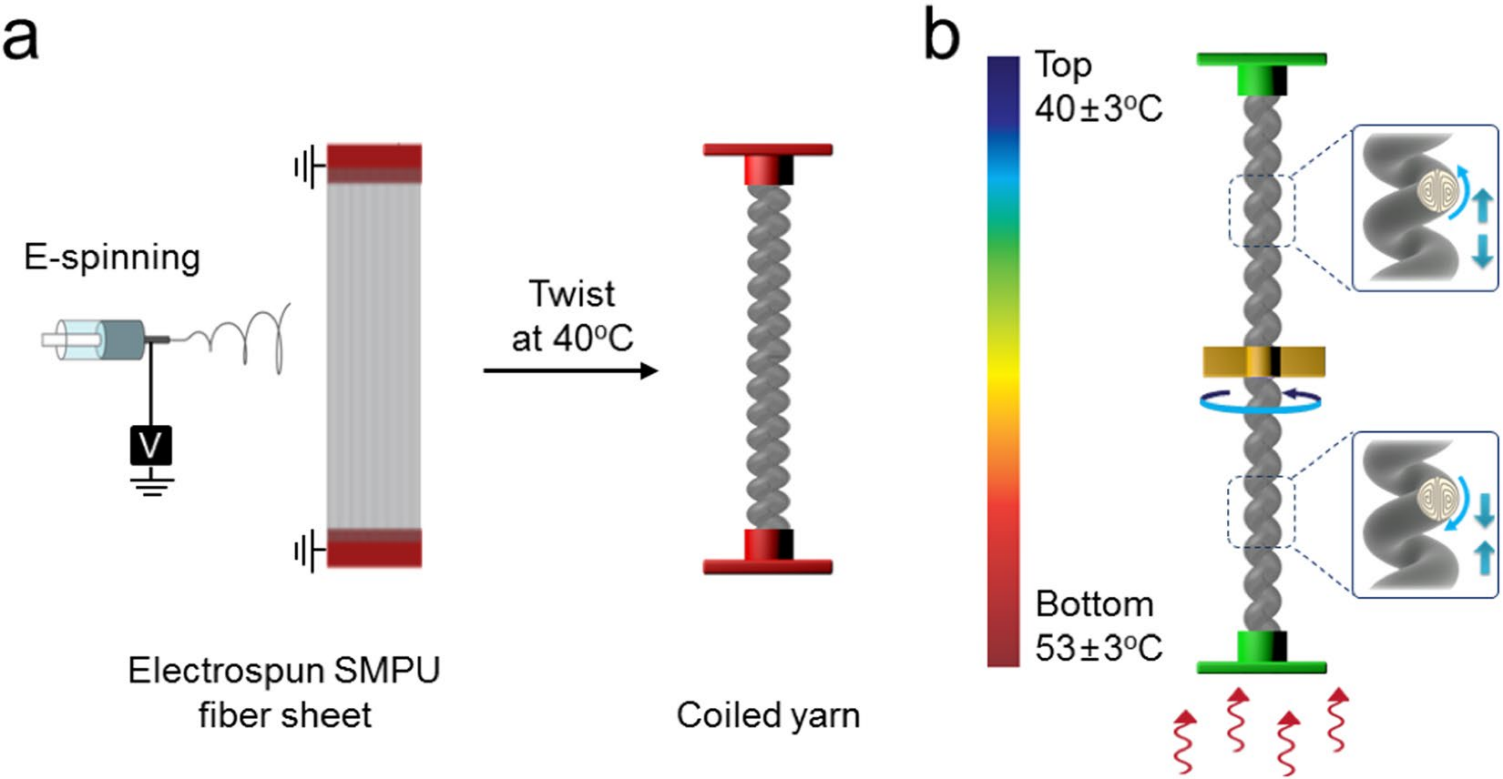
(**a**) Illustration of the fabrication of a coiled SMPU yarn torsional muscle and (**b**) the utilized muscle configuration and the oppositely directed length changes and twist of the hotter yarn segment (a contraction and untwist) and the colder yarn segment (an expansion and up twist) during torsional actuation. Both ends of a coiled SMPU yarn were attached to cantilever that prohibited end rotation, but allowed changes in total muscle length.

The SMPU yarn precursor was fabricated into a torsional muscle in two steps (Figs 1a and S1). Firstly, SMPU microfibers were prepared by electrospinning and collected as an aligned sheet^23^. The electrospinning processes tends to elongate and orient the hard and soft segments of the SMPU in the microfiber direction^24^, which is important for providing the anisotropic thermal expansion that is needed for high stroke torsional actuation. Secondly, the torsional muscle was fabricated by inserting twist into the electrospun SMPU microfiber sheet at 40 °C. The sheets were twisted until coils formed in the resulting yarn, and then further twisted until the entire yarn length was coiled. Cooling to room temperature fixed the twisted and coiled yarn shape. The yarn diameter described is the diameter before the onset of coiling and the inserted twist is relative to the length of the sheet of electrospun SMPU nanofibers.

The stretched muscle was torsionally tethered at opposite ends, but positionally tethered at only one end, and then exposed to a temperature gradient along its length (Fig. S2), which provided fast, giant stroke torsional actuation (Fig. 1b). A paddle attached at the muscle mid-point indicated the torsional actuation. The two-end-tethered torsionally configuration conserves the total twist (twist plus writhe) during torsional actuation of the muscle, but the temperature gradient along the length of the SMPU muscle and the resultant differences in thermal expansion allows for the symmetry breaking that enables torsional actuation. The hotter regions of the SMPU muscle untwist and the cooler regions up twist. With the temperature gradient shown in Fig. 1b, the result is paddle rotation in the untwist direction of the bottom, hotter part of SMPU muscle. This above described initial giant stroke is not used for electrical energy harvesting, which is instead due to convection-driven fluctuations in the average temperature difference between upper and bottom yarn segments.

### Torsional SMPU muscle powered by natural thermal convection

**Figure 2:**
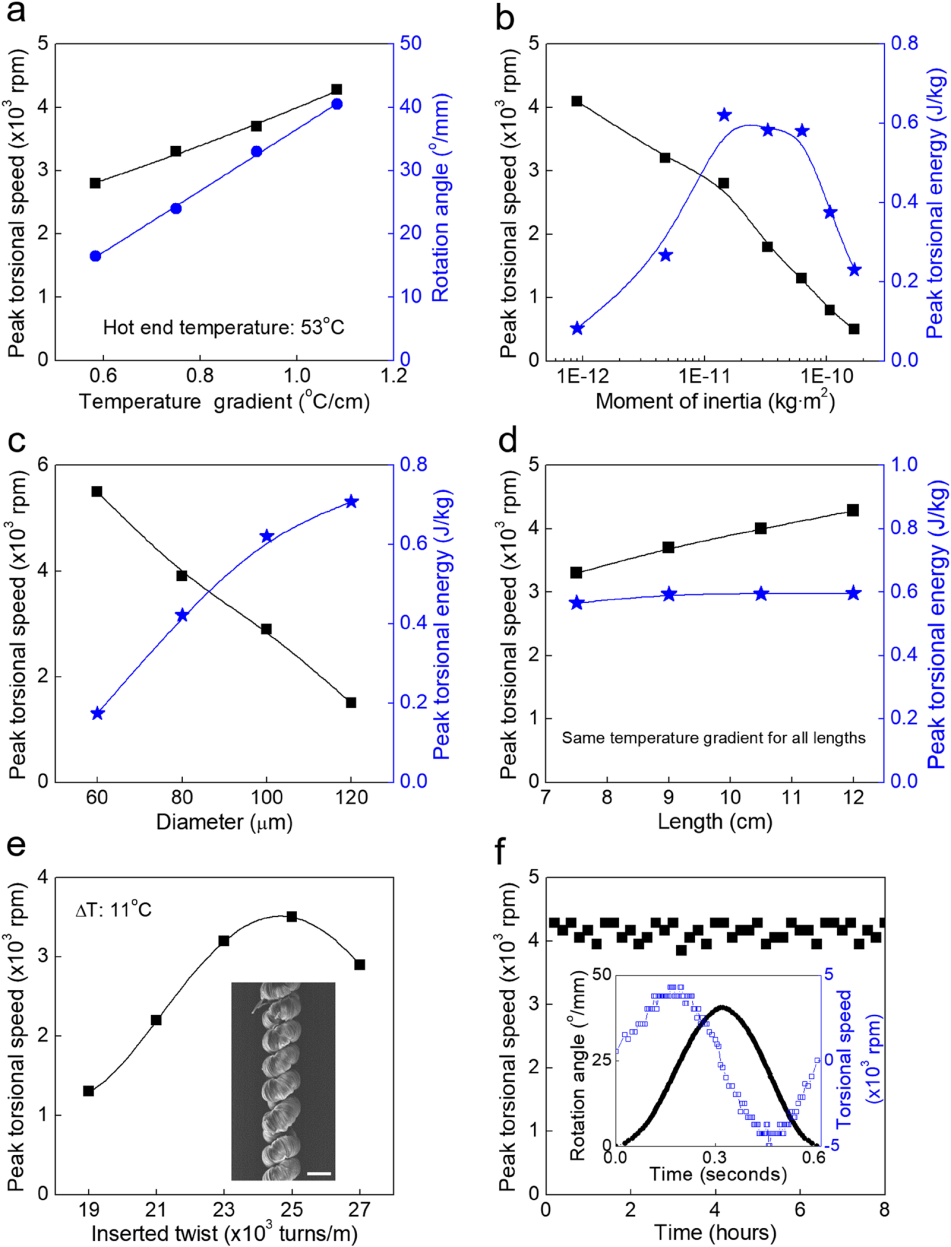
Torsional actuation of a two-end-tethered SMPU muscle that results from the initial temperature gradient produced by natural convection. Unless otherwise indicated, a 100-µm-diameter SMPU muscle with 12 cm stretched length and 25,000 turns/m inserted twist was used, temperature gradients are normalized with respect to the stretched muscle length, the hot end temperature was 53 °C, and the paddle’s moment of inertia was 8.9 × 10^−13^ kg·m^2^. (**a**) The dependence of peak torsional speed (black squares) and rotation angle (blue circles) on the average temperature gradient along the muscle length. (**b**) Peak torsional speed (black squares) and kinetic energy (blue stars) as a function of the moment of inertia of the paddle. (**c**) Peak torsional speed (black squares) and peak torsional kinetic energy (blue stars), optimized for the moment of inertia of paddle, as a function of the diameter of the SMPU muscle. The temperature gradient for (**b** and **c**) was 1.08 °C/cm. (**d**) Peak torsional paddle speed (black squares) and peak torsional paddle kinetic energy per muscle length (blue stars) as a function of muscle length for 50%-stretched muscles. The paddle used, which has a moment of inertia of 8.9 × 10^−13^ kg·m^2^, comprised a pair of disc-type NdFeB-N50 magnets. The temperature gradient was 1.08 °C/cm. (**e**) The dependence of peak torsional speed of a SMPU muscle on the degree of twist insertion for a temperature difference of 11 °C and a bottom temperature of 53 °C. Inset: SEM image of a fully-coiled SMPU muscle (scale bar: 100 μm). (**f**) An eight hour cycle test (for a 46 °C hot-end temperature) showing that the peak torsional speed does not significantly change with cycle time. Inset: rotation angle (black filled symbols) and torsional speed (blue open symbols) versus time during one cycle of untwist and retwist.

Since our ultimate aim is to rotate a permanent magnet, to thereby enable mechanical-to-electrical energy conversion, we first focused on maximizing the mechanical energy output from torsional actuation in response to the initial temperature gradient produced by convection above a heated plate. Not surprisingly, the peak torsional speed and torsional rotation angle were approximately proportional to the temperature gradient along the axial direction of the SMPU muscle (Fig. 2a).

These experiments used 100-µm-diameter SMPU yarn that had been stretched by 50% (from 8 cm to 12 cm) by applying a fixed force, and then exposed to temperature gradients with a fixed maximum temperature of about 53 °C (at the yarn end nearest to the heat source). The minimum temperature at the yarn end most distant from the heat source ranged from 40 °C to 46 °C. Pre-stretching the SMPU muscle yarns increased torsional speed, since the thereby opened-up coils could be heated more effectively and inter-coil contact does not hinder torsional rotation (Fig. S3). A rotor having a moment of inertia of 8.9 × 10^−13^ kg·m^2^ was used for the below described initial measurements. When the temperature difference between yarn ends was 13 °C (corresponding to a temperature gradient of 1.08 °C/cm, per length of the 50% stretched SMPU) the peak torsional speed was 4,280 rpm and the oscillation amplitude was 40°/mm (based on the full stretch length of the SMPU muscle). This torsional oscillation amplitude is large compared to the approximately 2°/mm torsional rotation observed for a twisted nylon-6 fiber that has been heated from 25 °C to 62 °C^25,26^. The torsional stroke was insensitive to the actual temperature of the hot end of the sample when the same temperature gradient was used, except when the hot end temperature was only slightly above the SMPU T_g_ of 31 °C (Fig. S4). The maximum torsional kinetic energy of the paddle attached to SMPU muscle was approximately 0.6 J/kg, when normalised to the total mass of actuating fibre. This kinetic energy was calculated as previously described^15^ from ½ *Iω*^2^, where *I* and *ω* are the moment of inertia and maximum angular velocity of the paddle, respectively.

Since the above relationship shows that the available kinetic energy depends upon the moment of inertia of the paddle, experiments were next conducted using paddles having different moments of inertia. Figure 2b shows the dependence of peak rotation speed and peak torsional kinetic energy on paddle moment of inertia for a paddle attached to a 100-μm-diameter coiled SMPU muscle. Similar data obtained for 60, 80 and 120-µm-diameter SMPU muscles are in Fig. S5. As expected, rotation speed decreased when the muscle yarn was attached to a paddle with a higher inertial moment. In addition, a peak in the paddle kinetic energy occurred at a particular paddle inertia that increased as yarn diameter increased (Fig. S5). Having a high kinetic energy at a particular moment of inertial paddle is different from previous torsional polymer muscles, which had constant kinetic energy regardless of the paddle’s moment of inertia^20^.

The peak kinetic energy was also found to be approximately proportional to the diameter of a SMPU muscle (Fig. 2c). Larger diameter yarns generate higher torques^27^ when slowly heated, suggesting higher rotation speeds should be generated by thicker yarns. However, the temperature fluctuations in the current work are likely to only heat the surface of the thicker yarns and the cooler inner yarn core restricts the torque and torsional rotation speeds. Hence, the problem of heat transfer limits the benefit of using very thick yarns.

The peak torsional speed of the SMPU muscle increased with increasing yarn length, but the paddle kinetic energy normalized to the length of SMPU muscle was essentially constant (Fig. 2d). Previous studies have shown that torsional stroke scales linearly with muscle yarn length^15^ and it is known that the period of a torsional oscillator increases as the square root of length. These two effects suggest that the average torsional speed for the first half cycle should increase with the square root of yarn length. Indeed, the peak torsional speeds shown in Fig. 2d do show this dependence, although the range of muscle lengths investigated was limited. Paddle torsional kinetic energy depends on the square of the torsional speed so it is expected that the paddle kinetic energy normalized per length of SMPU muscle would be largely independent of length, as observed (Fig. 2d).

Fully coiled muscles were fabricated with different amounts of inserted twist, with 25,000 turns/m showing highest performance (Fig. 2e). Reversible torsional actuation was maintained without loss in performance for 8 hours when driven by temperature fluctuations from a constant temperature gradient (Fig. 2f). The Fig. 2f inset shows the full reversibility realized for one cycle of untwisting and re-twisting a SMPU muscle.

### Electrical energy harvesting from natural thermal convection

Unlike previously work in which torsional actuation was controlled by electrical heating or a heat-gun^19,20^, the SMPU muscles were continuously driven by temperature fluctuations resulting from natural convection above a hot plate (which in practical applications could be a radiator for room heating). Fluctuations in air movement in a room caused fluctuations in the temperatures of muscle segments, causing torsional actuation whose mechanical energy was harvested as electrical energy. Since the top and bottom of the muscle are torsionally tethered, the direction of torque change on the magnetic rotor will depend that whether a fluctuation in natural convection increases or decreases the average temperature difference between top and bottom muscle segments.

**Figure 3:**
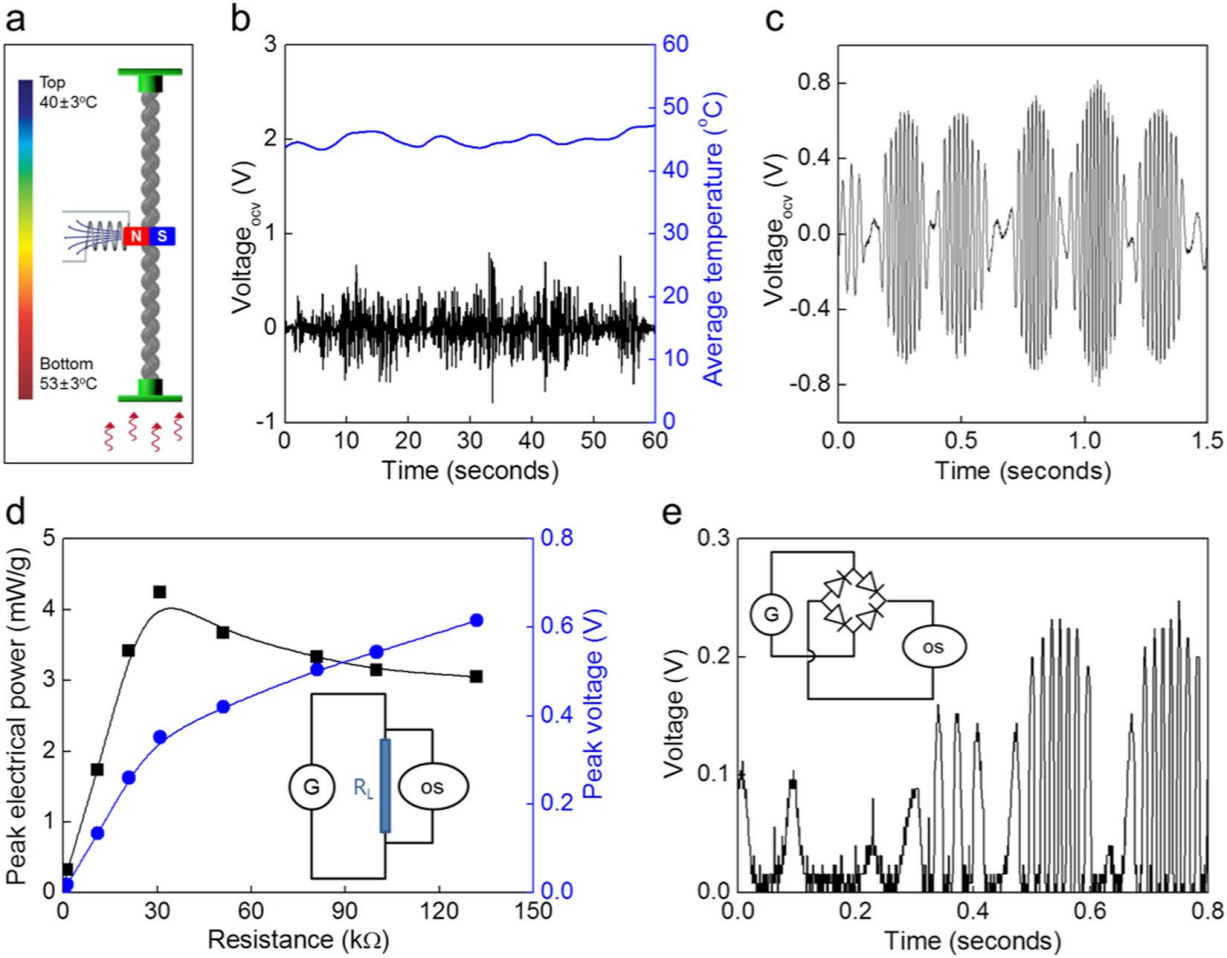
Harvesting thermal energy as electrical energy using torsional actuation of a SMPU muscle that is driven by natural convection. (**a**) Schematic illustration of the generator, which outputs electrical energy from the mechanical energy generated by the temperature gradient produced by natural convection. (**b**) The time dependence of the open circuit voltage on the electromagnet coils that is generated by muscle powered rotation of a magnet, when the hot-end temperature is 46 °C and the voltage is measured using an oscilloscope and a 1 MΩ impedance probe. The corresponding time dependence of the average of yarn top and yarn bottom temperatures is also shown. (**c**) Magnified view of the time dependence of generated open-circuit voltage). (**d**) The generated peak electrical power (black squares) and peak voltage (blue circles) as a function of the load resistance. Inset: illustration of the configuration used for impedance matching. (**e**) The voltage output after rectification by a bridge diode. Inset: The circuit for rectifying the voltage.

Electrical energy harvesting by using temperature fluctuations associated with natural thermal convection was investigated for a torsional muscle that was optimized to provide maximum rotor kinetic energy (Fig. 3a). The generator comprised a neodymium magnet rotor, two copper wire coils, and a 100-μm-dimeter SMPU muscle that was 12-cm-long (after stretching 50%) (Fig. S6). The rotor comprised a stack of three magnets, which were selected to match the optimized moment of inertia (1.45 × 10^−11^ kg·m^2^ from Fig. 2b) and were attached to the midpoint of the muscle. When the hot end temperature of the SMPU muscle was 53 °C and temperature difference between yarn ends was 13 °C, the peak torsional speed of the magnet rotor was 3,000 rpm (Fig. 3b and movie S1). The resulting maximum open circuit voltage from the generator was 0.81 V (Fig. 3b,c). Under these experimental conditions, and an impedance matched load 31 kΩ, the generator produced a peak power output of 4.0 W/kg and an energy per cycle of 0.43 J/kg when normalized by the mass of the SMPU muscle (Fig. 3d). This peak power density is similar to the thermoelectric power generated by a temperature difference of 10 °C^28^. Based on the volume of SMPU muscle, the volumetric peak power output and energy per cycle were 2.20 mW/cm^3^ and 0.24 mJ/cm^3^, respectively. Based on the total volume of the electromagnetic generator, the magnetic rotor, and the SMPU muscle, including all void space, the volumetric peak power and energy per cycle were 0.595 μW/cm^3^ and 0.064 μJ/cm^3^, respectively.

The efficiency of conversion of torsional kinetic energy into electrical energy was 9.3%, which was calculated using equation (1):

1 $${\rm{Efficiency}}\,(\eta )=\frac{{\int }_{{{\rm{t}}}_{1}}^{{{\rm{t}}}_{2}}{{\rm{V}}}^{2}/{\rm{R}}\,{\rm{dt}}}{{\sum }_{{\rm{n}}=1}^{{\rm{n}}}\frac{1}{2}I\cdot {{\rm{\omega }}}_{{\rm{n}}}^{2}}$$

In this equation V is the generated voltage with external resistor *R*, *I* is the moment of inertia, and ω_n_ is the n^th^ peak torsional angular velocity between t_1_ and t_2_. The generated AC voltage can be rectified using a commercial bridge rectifier, as shown in Fig. 3e. The rectified voltage was 0.28 V, which was smaller than the peak generated voltage due to the voltage drop across the bridge rectifier.

## Discussion

A previously reported thermal energy harvester using NiTi shape memory alloy (SMA) wire provides a useful benchmark^13^. The SMA wire rotated on pulleys when hot and cold sinks were applied. When normalized to the weight of the 300-µm-diameter NiTi SMA wire, an average electrical power density of 14.2 W/kg was obtained when the temperature difference between hot and cold baths was 50 °C. When a 0.7-mm-thick SMA sheet belt was used^14^, the average output power per SMA weight was 4.43 W/kg for a temperature difference between hot and cold baths of 51 °C. Since, tensile actuation of NiTi is highly hysteretic, they could not be used to harvest the energy due to the presently investigated minute changes of temperature. Another limitation of SMA wires are their high cost (~$3,400/kg)^29^ which is ~70 times higher than SMPU (~$50/kg).

In summary, we have demonstrated a continuously operating, oscillating torsional artificial muscle driven by the small temperature gradients typically encountered in daily-life. The energy harvester used commercially-available SMPU, and effectively harvests the natural convection to produce 4.0 W/kg of peak output electrical energy. The muscle can be easily fabricated by twist insertion and scaled up for increasing the amount of generated electrical energy by increasing muscle diameter and length. This novel energy harvesting system can be applied various places where natural convection occurs. While the weight and occupied volume of the electromagnetic generator is presently massive compared with that of the yarn muscle, alternative means, like triboelectric generators^30^, are being pursued to more suitably size the thermal energy harvesting muscle to the mechanical energy harvester.

## Method

### Materials

The SMPU shape memory polymer (MM-2520) and tetrahydrofuran were purchased from Technologies Inc. (Japan) and Aldrich (USA), respectively.

### Preparation of SMPU muscle

The SMPU microfibers were electrospun from a 5.5 wt% solution of SMPU in tetrahydrofuran, which by was made by stirring the polymer powder in the solvent for 7 days at room temperature. Sheets comprising 2-μm-diameter SMPU microfibers were made by an electrospinning process that is conventional, except for the use of grounded electrodes to provide microfiber alignment. The SMPU solution was fed at a rate of 13 μl/min by a syringe pump at an applied voltage of 18 kV between the syringe needle (+11 kV) and the collector (–7 kV) using a high-voltage DC power supply (Wookyong TECH, Korea). The distance between the syringe needle and the collector was 20 cm. Opposite ends of the SMPU sheet were attached to an electric motor shaft with a flat rectangular paddle and a fixed support, respectively. Using the rotating paddle, the SMPU sheet was twisted at 40 °C until the thereby produced SMPU yarn was fully-coiled.

### Analysis of torsional speed and stroke

Two methods were deployed for measurement of thermally driven torsional actuation: (1) Frame-by-frame analysis of movies taken using a high-speed camera (1,000 frames/s, Phantoms, Video S1) and (2) analysis of the voltage signal from the energy harvesting system using an oscilloscope. The number of peaks and the frequency of the time trace of the voltage signal correspond to the number of rotations and the rotation speed of SMPU muscle, respectively.

### Characterization

We used scanning electron microscope (SEM) images (FE-SEM, Hitachi S4700) to characterize yarn morphology. The thermal properties of the SMPU yarn were measured using a dynamic mechanical analyzer (Seiko Exstar 6000).

## Electronic supplementary material

Movie S1

Supporting Information
