# Supplementary material for: Harvesting electrical energy from torsional thermal actuation driven by natural convection
Source: Sci Rep. 2018 Jun 7;8:8712. doi: 10.1038/s41598-018-26983-4 (PMC5992175; doi:10.1038/s41598-018-26983-4)
Supplement: Supplementary file 2 — Supporting Information [file 41598_2018_26983_MOESM2_ESM.docx]

Supporting Information

Harvesting electrical energy from torsional thermal actuation driven by natural convection

*Shi Hyeong Kim^1^, Hyeon Jun Sim^1^, Jae Sang Hyeon^1^, Dongseok Suh^2^, Geoffrey M. Spinks^3^, Ray H. Baughman^4^ & Seon Jeong Kim^1^*

^1^Center for Self-powered actuator and Department of Biomedical Engineering, Hanyang University, Seoul 133-791, South Korea.

[*] E-mail: [sjk@hanyang.ac.kr](mailto:sjk@hanyang.ac.kr)

^2^Dept. of Energy Science, Sungkyunkwan University, Suwon, Gyeonggido 16419, South Korea.

^3^Intelligent Polymer Research Institute, ARC Centre of Excellence for Electromaterials Science, University of Wollongong, Wollongong, New South Wales 2522, Australia.

^4^The Alan G. MacDiarmid NanoTech Institute, University of Texas at Dallas, Richardson, TX 75083, USA.


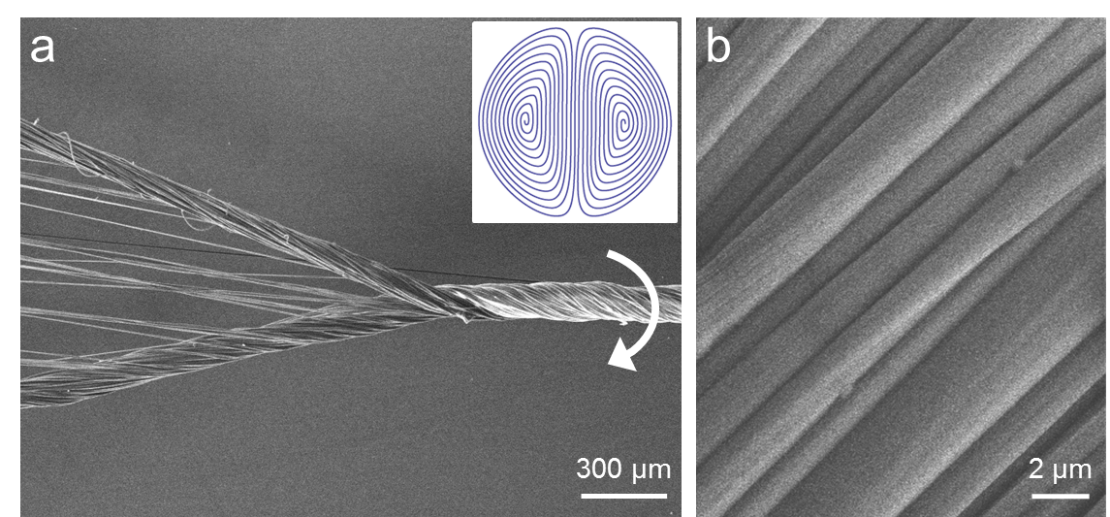


**Figure S1.** (a) SEM image of twisting a SMPU microfiber sheet into a yarn having a dual-Archimedean structure. Inset: Illustration of the cross-section of a dual-Archimedean yarn. (b) SEM image of the side-wall of a 140-μm-diameter SMPU yarn. The white arrow indicates the yarn direction.


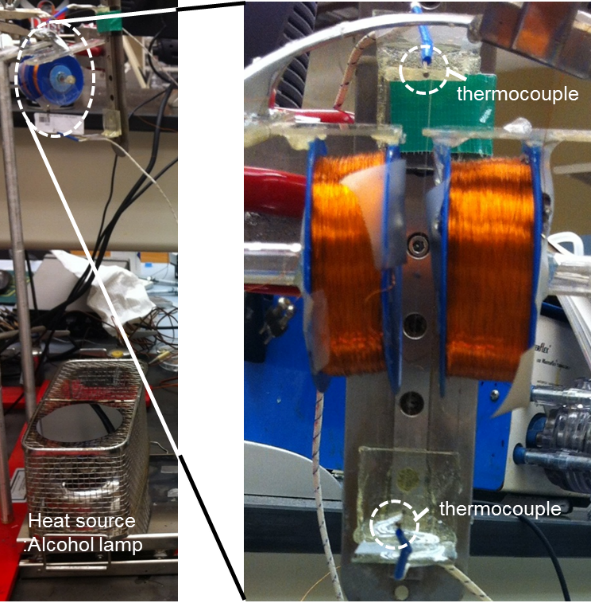


**Figure S2.** Photograph showing the experimental apparatus. Temperatures of opposite ends of the-SMPU yarn were measured using thermocouples.


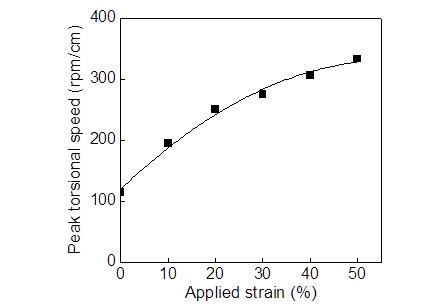


**Figure S3.** The strain dependence of a peak torsional speed of a 100-µm-diameter coiled SMPU yarn for a temperature gradient per yarn length of 0.91^o^C/cm (normalized to the non-stretched yarn length) and a bottom temperature of 53^o^C. The moment of inertia of the paddle was 1.45x10^-11^ kg⋅m^2^. Here and elsewhere, the yarn diameter mentioned is the diameter before stretching.


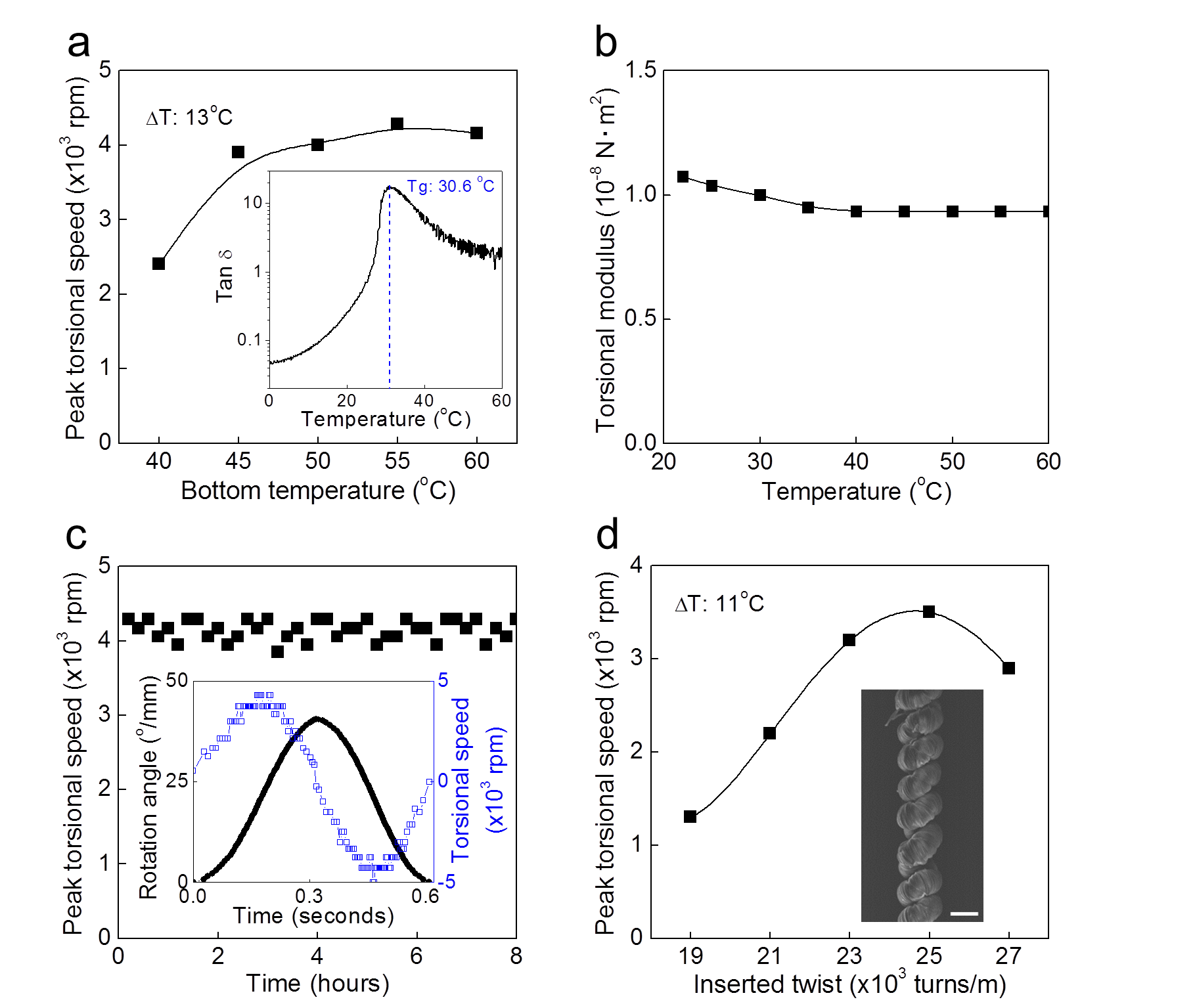


**Figure S4.** Torsional actuation speed for a hot-end to cold-end temperature difference of 13^o^C (corresponding to an average temperature gradient of 1.08^o^C/cm, per length of SMPU yarn) as a function of hot-end temperature. Inset: Dynamic mechanical analysis results showing the SMPU microfibers in the twisted yarn have a glass transition temperature of 30.6^o^C.


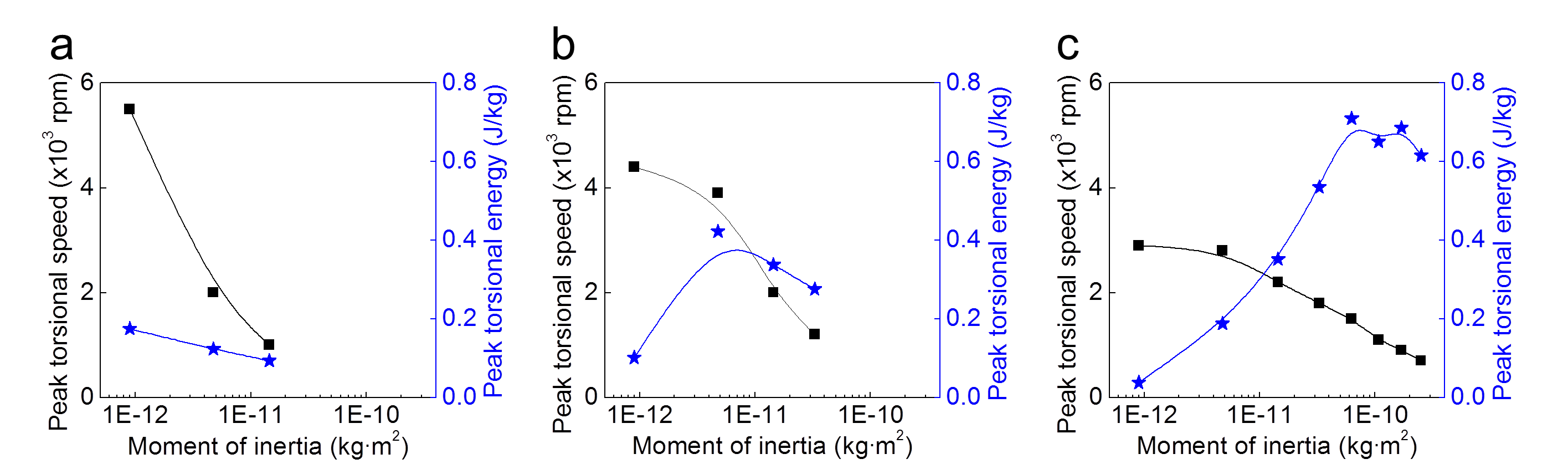


**Figure S5.** Peak torsional speed (black squares) and kinetic energy (blue stars) as a function of the moment of inertia of the paddle for yarn diameters of (a) 60, (b) 80, and (c) 120 µm. These results are for a hot-side to cold-side temperature difference of 13^o^C and a hot side temperature of 53^o^C (corresponding to a temperature gradient of 1.08^o^C/cm, per length of SMPU yarn). The moment of inertia was controlled by the varying the number of disk-shaped magnets that were stacked to form the rotor. The magnets were NdFeB-N50 (from Excelpoint Inc, Korea), which each weighed 2.85 mg and had a height of 0.5 mm and a diameter of 1 mm.


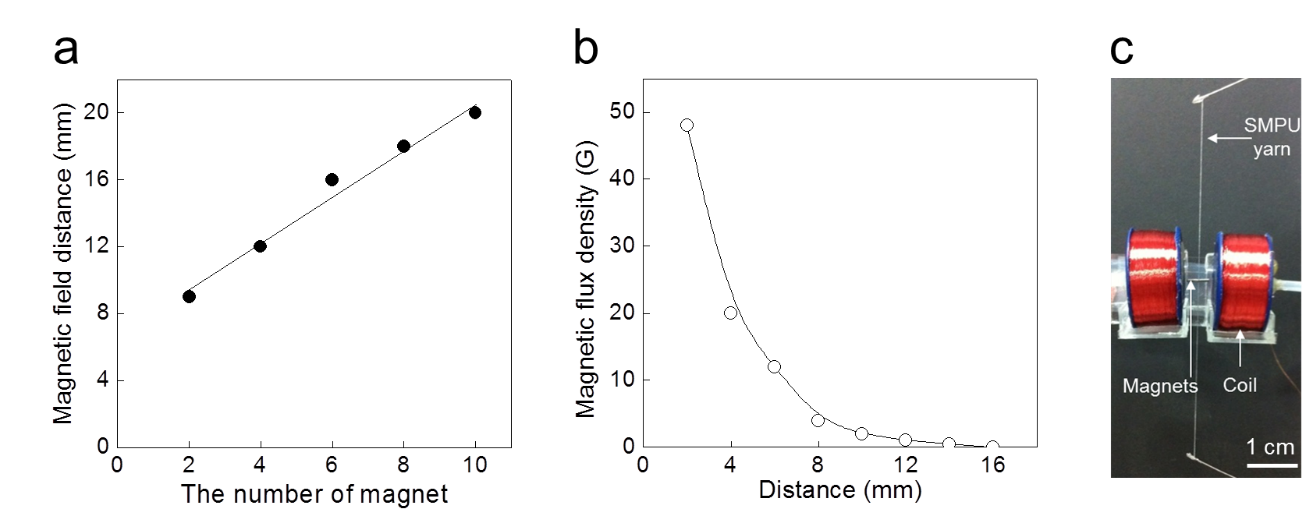


**Figure S6. Results used for optimizing the coils of the electromagnetic generator.** (a) Separation between the magnetic rotor and the surrounding wire coils depending upon the number of 0.5 mm thick magnets in the magnet stack that forms the rotor. (b) The magnetic flux density from a 6 magnets stack as a function of the measurement distance. (c) Photograph showing the generator system. Considering three magnet pairs in the rotor, each coil was made by winding 30,000 turns of an 100-µm-diameter enamel coated copper wire. The outer diameter, inner diameter, and length of coils were 18, 5, and 11 mm, respectively. The magnets used for the rotor were 1-mm-diameter, 0.5 mm high NdFeB-N50 disks. The number in N50 indicates the maximum energy product in Mega-Gauss Oersteds (MGOe).

**Movie S1.** Movie showing thermal-convection-driven torsional actuation of a coiled SMPU yarn muscle. Even though the temperature difference between hot and cold yarn ends was only 13^o^C, the rotor (comprising three pairs of stacked magnets) rotated at a peak speed of 3000 rpm. The bottom temperature was 53^o^C. The torsional muscle was 100 μm in yarn diameter and 12-cm-long (after stretching 50%).
